# 3-(3-Bromo-4-methoxy­phen­yl)-1,5-diphenyl­pentane-1,5-dione

**DOI:** 10.1107/S1600536810008548

**Published:** 2010-03-13

**Authors:** Grzegorz Dutkiewicz, C. S. Chidan Kumar, H. S. Yathirajan, B. Narayana, Maciej Kubicki

**Affiliations:** a Department of Chemistry, Adam Mickiewicz University, Grunwaldzka 6, 60-780 Poznań, Poland; b Department of Studies in Chemistry, University of Mysore, Manasagangotri, Mysore 570 006, India; c Department of Studies in Chemistry, Mangalore University, Manasagangotri, Mangalagangotri 574 199, India

## Abstract

In the title compound, C_24_H_21_BrO_3_, the central bromo­methoxy­benzene ring forms dihedral angles of 63.6 (1) and 60.3 (1)° with the terminal phenyl rings, while the angle between the two phenyl rings is 25.8 (1)°. The crystal structure is stabilized by weak C—H⋯Br and C—H⋯O hydrogen bonds, and C—H⋯π and π–π stacking [centroid–centroid distance = 3.910 (3) Å] inter­actions.

## Related literature

For 1,5-diketones, see: Hirsch & Bailey (1978 ▶). For related structures, see: Das *et al.* (1994 ▶); He *et al.* (2008 ▶); Li *et al.* (2008 ▶); Teh *et al.* (2006 ▶). For a description of the Cambridge Structural Database, see: Allen (2002 ▶).
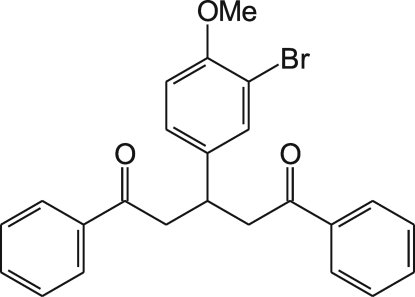


## Experimental

### 

#### Crystal data

C_24_H_21_BrO_3_
*M* *_r_* = 437.32
Monoclinic, 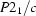

*a* = 12.7305 (4) Å
*b* = 7.14024 (19) Å
*c* = 22.8133 (8) Å
β = 105.602 (3)°
*V* = 1997.28 (11) Å^3^
*Z* = 4
Mo *K*α radiation
μ = 2.08 mm^−1^
*T* = 100 K
0.5 × 0.5 × 0.3 mm

#### Data collection

Oxford Diffraction Xcalibur Eos CCD diffractometer
Absorption correction: multi-scan (*CrysAlis PRO*; Oxford Diffraction, 2009 ▶) *T*_min_ = 0.471, *T*_max_ = 0.536
7645 measured reflections
4094 independent reflections
3289 reflections with *I* > 2σ(*I*)
*R*_int_ = 0.016

#### Refinement

*R*[*F* ^2^ > 2σ(*F* ^2^)] = 0.024
*wR*(*F* ^2^) = 0.057
*S* = 1.00
4094 reflections
254 parameters
H-atom parameters constrained
Δρ_max_ = 0.34 e Å^−3^
Δρ_min_ = −0.36 e Å^−3^

### 

Data collection: *CrysAlis PRO* (Oxford Diffraction, 2009 ▶); cell refinement: *CrysAlis PRO*; data reduction: *CrysAlis PRO*; program(s) used to solve structure: *SIR92* (Altomare *et al.*, 1993 ▶); program(s) used to refine structure: *SHELXL97* (Sheldrick, 2008 ▶); molecular graphics: *ORTEP-3* (Farrugia, 1997 ▶) and *Mercury* (Macrae *et al.*, 2008 ▶); software used to prepare material for publication: *Stereochemical Workstation Operation Manual* (Siemens, 1989 ▶) and *SHELXL97*.

## Supplementary Material

Crystal structure: contains datablocks I, global. DOI: 10.1107/S1600536810008548/is2528sup1.cif

Structure factors: contains datablocks I. DOI: 10.1107/S1600536810008548/is2528Isup2.hkl

Additional supplementary materials:  crystallographic information; 3D view; checkCIF report

## Figures and Tables

**Table 1:** Hydrogen-bond geometry (Å, °) *Cg*1 is the centroid of the C31–C36 ring.

*D*—H⋯*A*	*D*—H	H⋯*A*	*D*⋯*A*	*D*—H⋯*A*
C13—H13*A*⋯Br33^i^	0.95	2.76	3.613 (2)	149
C35—H35*A*⋯O1^ii^	0.95	2.37	3.245 (2)	153
C36—H36*A*⋯O5^iii^	0.95	2.56	3.493 (2)	168
C54—H54*A*⋯*Cg*1^iv^	0.95	2.60	3.489 (3)	155

Symmetry codes: (i) 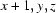; (ii) 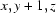; (iii) 
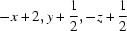
; (iv) 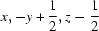.

**Fig. 1.**
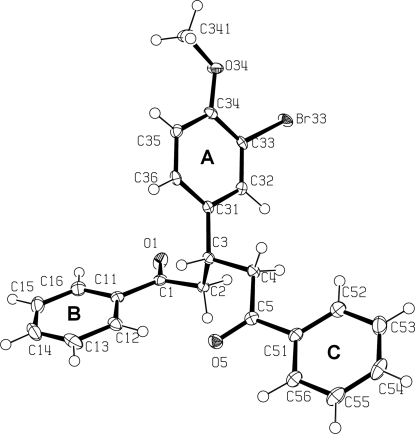
The structure of (I), showing 50% probability displacement ellipsoids and the atom-numbering scheme.

**Fig. 2.**
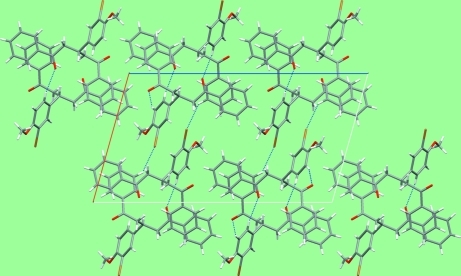
The crystal packing of (I) viewed along the b axis. Hydrogen bonds are shown as dashed lines.

## supplementary crystallographic information

### Comment

1,5-Diketones are important synthetic intermediates and starting materials in the synthesis of many heterocyclic compounds (e.g., Hirsch & Bailey, 1978). The related 3-aryl-derivatives of 1,5-diarylopentano-1,5-dione can be also regarded, due to the conformational flexibility and the relative easiness of introducing different substituents, as an interesting group of compounds for studying the factors influencing molecular conformation and intermolecular interactions. Several structures have been already determined, for instance the non-substituted 1,3,5-triphenyl-1,5-pentanedione (Das *et al.*, 1994), 3-(4-dimethylaminophenyl)-1,5-diphenylpentane-1,5-dione (He *et al.*, 2008) or 1,5-bis(4-chlorophenyl)-3-(2,5-dimethoxyphenyl)pentane-1,5-dione (Teh *et al.*, 2006). We present here the crystal structure of another simple 1,5-diphenyl-1,5-diketone derivative, 3-(3-bromo-4-methoxyphenyl)-1,5-diphenylpentane-1,5-dione (I, Scheme 1).

The overall conformation of (I) might be described by the dihedral angles between the approximately planar aromatic fragments. The bromomethoxybenzene ring (A, Fig. 1) in (I) forms dihedral angles of 63.6 (1) and 60.3 (1)° with the terminal phenyl rings B and C, respectively, and the rings B and C, in turn, make the dihedral angle of 25.8 (1)°. In the similar structures found in the Cambridge Crystallographic Database (Allen, 2002) there is no clear preference for any type of overall conformation, the dihedral angles cover wide range of values. The same is true for the conformation of the central C_5_-chain which can be almost extended [as for instance in 1,5-bis(4-bromophenyl)-3-phenyl-pentane-1,5-dione; Li *et al.*, 2008], or is more folded as in (I), where the torsion angles along the C_5_ chain are -70.7 (2), 174.7 (2), -74.4 (2) and 179.9 (1)°. The common feature for all similar structures, also observed in (I), is the coplanarity of the keto-O atoms with the adjacent phenyl rings. In (I) the deviations from the mean planes are 0.152 (3) Å for O1 and 0.050 (3) Å for O5.

In the crystal structure there is a weak C13—H13···Br33(*1+x, y, z*) contact [H···Br distance 2.81 (2) Å, C—H···Br angle 143 (2)°] that links molecules into infinite chains along the *x *direction. Two weak C—H···O contacts, C35—H35···O1(*x, 1+y, z*) and C36—H36···O5(*2-x, 0.5+y, 0.5-z*), with H···O distances of 2.37 and 2.56 Å, respectively, link molecules into infinite chains along the *y *direction. An additional weak C—H···π contact [C54—H54···*Cg*1(*x, 0.5-y, 0.5+z*); *Cg*1 is the centroid of ring A] and a π–π stacking interaction between rings B and C stabilize the packing. For this latter interaction, the centroid-centroid distance is 3.910 (3) Å, an interplanar distance is 3.505 Å with a relatively large offset (the overlap is partial only) - 1.73 Å.

### Experimental

Acetophenone (2.40 g, 0.02 mol) was mixed with 3-bromo-4-methoxybezaldehyde (2.15 g, 0.01 mol) and dissolved in ethanol (50 ml). To this, 5 ml of KOH (50%) was added. The reaction mixture was stirred for 8 hours. The resulting crude solid was filtered, washed successively with distilled water and finally recrystallized from ethanol (95%) to give the pure compound. Crystals suitable for X-ray diffraction studies were grown by slow evaporation of an acetone solution (m.p. 381 K).

### Refinement

The H atoms were placed in idealized positions and constrained to ride on their parent atoms, with C—H = 0.95 Å with *U*_iso_(H) = 1.2*U*_eq_(C) for phenyl hydrogen; 0.98 Å with *U*_iso_(H) = 1.5*U*_eq_(C) for CH_3_ group; 0.99 Å with *U*_iso_(H) = 1.2*U*_eq_(C) for CH_2_ group; 1.00 Å with *U*_iso_(H) = 1.2*U*_eq_(C) for CH group.

### Crystal data

**Table:**

C_24_H_21_BrO_3_	*F*(000) = 896
*M**_r_* = 437.32	*D*_x_ = 1.454 Mg m^−^^3^
Monoclinic, *P*2_1_/*c*	Mo *K*α radiation, λ = 0.71073 Å
Hall symbol: -P 2ybc	Cell parameters from 5038 reflections
*a* = 12.7305 (4) Å	θ = 3.0–28.0°
*b* = 7.14024 (19) Å	µ = 2.08 mm^−^^1^
*c* = 22.8133 (8) Å	*T* = 100 K
β = 105.602 (3)°	Block, yellow
*V* = 1997.28 (11) Å^3^	0.5 × 0.5 × 0.3 mm
*Z* = 4	

### Data collection

**Table:**

Oxford Diffraction Xcalibur Eos CCD diffractometer	4094 independent reflections
Radiation source: fine-focus sealed tube	3289 reflections with *I* > 2σ(*I*)
graphite	*R*_int_ = 0.016
Detector resolution: 16.1544 pixels mm^-1^	θ_max_ = 28.1°, θ_min_ = 3.0°
ω scan	*h* = −12→16
Absorption correction: multi-scan (*CrysAlis PRO*; Oxford Diffraction, 2009)	*k* = −8→8
*T*_min_ = 0.471, *T*_max_ = 0.536	*l* = −25→27
7645 measured reflections	

### Refinement

**Table:**

Refinement on *F*^2^	Primary atom site location: structure-invariant direct methods
Least-squares matrix: full	Secondary atom site location: difference Fourier map
*R*[*F*^2^ > 2σ(*F*^2^)] = 0.024	Hydrogen site location: inferred from neighbouring sites
*wR*(*F*^2^) = 0.057	H-atom parameters constrained
*S* = 1.00	*w* = 1/[σ^2^(*F*_o_^2^) + (0.0327*P*)^2^] where *P* = (*F*_o_^2^ + 2*F*_c_^2^)/3
4094 reflections	(Δ/σ)_max_ = 0.001
254 parameters	Δρ_max_ = 0.34 e Å^−^^3^
0 restraints	Δρ_min_ = −0.36 e Å^−^^3^

### Special details

**Table:**

Geometry. All s.u.'s (except the s.u. in the dihedral angle between two l.s. planes) are estimated using the full covariance matrix. The cell s.u.'s are taken into account individually in the estimation of s.u.'s in distances, angles and torsion angles; correlations between s.u.'s in cell parameters are only used when they are defined by crystal symmetry. An approximate (isotropic) treatment of cell s.u.'s is used for estimating s.u.'s involving l.s. planes.
Refinement. Refinement of *F*^2^ against ALL reflections. The weighted *R*-factor wR and goodness of fit *S* are based on *F*^2^, conventional *R*-factors *R* are based on *F*, with *F* set to zero for negative *F*^2^. The threshold expression of *F*^2^ > 2σ(*F*^2^) is used only for calculating *R*-factors(gt) etc. and is not relevant to the choice of reflections for refinement. *R*-factors based on *F*^2^ are statistically about twice as large as those based on *F*, and *R*- factors based on ALL data will be even larger.

### Fractional atomic coordinates and isotropic or equivalent isotropic displacement parameters (Å^2^)

**Table:**

	*x*	*y*	*z*	*U*_iso_*/*U*_eq_	
C1	0.91462 (13)	0.0724 (2)	0.35970 (8)	0.0145 (4)	
O1	0.86814 (9)	0.01848 (17)	0.39695 (5)	0.0217 (3)	
C2	0.85537 (13)	0.0764 (2)	0.29248 (7)	0.0134 (4)	
H2A	0.7862	0.0060	0.2862	0.016*	
H2B	0.9006	0.0104	0.2698	0.016*	
C3	0.82916 (13)	0.2738 (2)	0.26526 (8)	0.0131 (4)	
H3A	0.8993	0.3445	0.2726	0.016*	
C4	0.77919 (13)	0.2605 (3)	0.19628 (8)	0.0145 (4)	
H4A	0.7216	0.1633	0.1880	0.017*	
H4B	0.7440	0.3815	0.1815	0.017*	
O5	0.95755 (9)	0.19514 (16)	0.18610 (5)	0.0191 (3)	
C5	0.86066 (14)	0.2138 (2)	0.16057 (8)	0.0145 (4)	
C11	1.03092 (13)	0.1341 (2)	0.37984 (8)	0.0149 (4)	
C12	1.08947 (14)	0.1805 (2)	0.33810 (9)	0.0185 (4)	
H12A	1.0549	0.1775	0.2957	0.022*	
C13	1.19792 (14)	0.2310 (3)	0.35891 (10)	0.0261 (5)	
H13A	1.2377	0.2622	0.3305	0.031*	
C14	1.24952 (15)	0.2366 (3)	0.42059 (10)	0.0296 (5)	
H14A	1.3241	0.2716	0.4344	0.035*	
C15	1.19147 (15)	0.1907 (2)	0.46233 (10)	0.0294 (5)	
H15A	1.2262	0.1945	0.5047	0.035*	
C16	1.08349 (14)	0.1398 (2)	0.44186 (9)	0.0209 (4)	
H16A	1.0442	0.1082	0.4705	0.025*	
C31	0.75354 (13)	0.3834 (2)	0.29399 (7)	0.0121 (4)	
C32	0.64779 (13)	0.3198 (2)	0.28958 (7)	0.0137 (4)	
H32A	0.6240	0.2031	0.2706	0.016*	
C33	0.57781 (12)	0.4268 (2)	0.31288 (7)	0.0125 (4)	
Br33	0.435504 (13)	0.33429 (2)	0.306668 (9)	0.01938 (6)	
O34	0.53210 (9)	0.69501 (16)	0.36041 (6)	0.0191 (3)	
C34	0.60856 (13)	0.5983 (2)	0.34074 (8)	0.0139 (4)	
C35	0.71445 (13)	0.6593 (2)	0.34608 (8)	0.0145 (4)	
H35A	0.7388	0.7746	0.3659	0.017*	
C36	0.78462 (13)	0.5528 (2)	0.32272 (7)	0.0138 (4)	
H36A	0.8564	0.5976	0.3266	0.017*	
C51	0.81896 (14)	0.1985 (2)	0.09296 (8)	0.0155 (4)	
C52	0.70940 (15)	0.2171 (2)	0.06268 (8)	0.0209 (4)	
H52A	0.6576	0.2364	0.0853	0.025*	
C53	0.67473 (16)	0.2080 (3)	−0.00028 (9)	0.0266 (5)	
H53A	0.5995	0.2212	−0.0207	0.032*	
C54	0.75009 (16)	0.1794 (2)	−0.03336 (8)	0.0254 (4)	
H54A	0.7267	0.1756	−0.0766	0.031*	
C55	0.85911 (16)	0.1566 (2)	−0.00367 (8)	0.0238 (4)	
H55A	0.9103	0.1338	−0.0264	0.029*	
C56	0.89418 (15)	0.1669 (2)	0.05928 (8)	0.0187 (4)	
H56A	0.9694	0.1525	0.0795	0.022*	
C341	0.55832 (15)	0.8827 (2)	0.38057 (10)	0.0272 (5)	
H34D	0.4945	0.9417	0.3891	0.041*	
H34A	0.5795	0.9535	0.3488	0.041*	
H34B	0.6189	0.8817	0.4177	0.041*	

### Atomic displacement parameters (Å^2^)

**Table:**

	*U*^11^	*U*^22^	*U*^33^	*U*^12^	*U*^13^	*U*^23^
C1	0.0172 (9)	0.0083 (8)	0.0187 (9)	0.0011 (7)	0.0057 (7)	−0.0009 (7)
O1	0.0261 (7)	0.0242 (7)	0.0172 (7)	−0.0093 (6)	0.0099 (5)	−0.0017 (6)
C2	0.0119 (8)	0.0158 (9)	0.0136 (9)	−0.0011 (7)	0.0052 (7)	−0.0013 (7)
C3	0.0104 (8)	0.0148 (8)	0.0149 (9)	−0.0019 (7)	0.0046 (7)	−0.0002 (7)
C4	0.0133 (8)	0.0162 (9)	0.0144 (9)	0.0002 (8)	0.0041 (7)	0.0009 (8)
O5	0.0147 (6)	0.0265 (7)	0.0168 (6)	0.0010 (6)	0.0056 (5)	0.0000 (5)
C5	0.0168 (9)	0.0110 (8)	0.0162 (9)	−0.0015 (7)	0.0055 (7)	0.0010 (7)
C11	0.0162 (9)	0.0073 (8)	0.0202 (9)	0.0018 (7)	0.0034 (7)	0.0000 (7)
C12	0.0156 (9)	0.0141 (9)	0.0254 (10)	0.0021 (8)	0.0047 (7)	0.0020 (8)
C13	0.0158 (9)	0.0182 (10)	0.0455 (13)	0.0037 (8)	0.0104 (9)	0.0059 (10)
C14	0.0136 (9)	0.0190 (10)	0.0499 (14)	−0.0001 (9)	−0.0020 (9)	−0.0038 (10)
C15	0.0257 (11)	0.0205 (11)	0.0324 (12)	0.0047 (9)	−0.0086 (9)	−0.0059 (9)
C16	0.0225 (10)	0.0132 (9)	0.0254 (10)	0.0022 (8)	0.0036 (8)	0.0002 (8)
C31	0.0105 (8)	0.0152 (9)	0.0104 (8)	−0.0008 (7)	0.0026 (6)	0.0017 (7)
C32	0.0143 (8)	0.0137 (8)	0.0127 (8)	−0.0026 (8)	0.0031 (7)	−0.0010 (7)
C33	0.0068 (8)	0.0161 (9)	0.0142 (9)	−0.0004 (7)	0.0020 (6)	0.0055 (7)
Br33	0.00941 (9)	0.01879 (10)	0.03102 (11)	−0.00150 (8)	0.00730 (7)	−0.00147 (9)
O34	0.0175 (6)	0.0152 (6)	0.0267 (7)	0.0022 (5)	0.0098 (5)	−0.0034 (5)
C34	0.0131 (8)	0.0156 (8)	0.0131 (9)	0.0035 (8)	0.0040 (7)	0.0037 (7)
C35	0.0157 (8)	0.0118 (8)	0.0150 (9)	−0.0011 (8)	0.0028 (7)	−0.0003 (7)
C36	0.0094 (8)	0.0177 (9)	0.0141 (9)	−0.0036 (7)	0.0028 (7)	0.0021 (7)
C51	0.0200 (9)	0.0114 (9)	0.0152 (9)	−0.0009 (7)	0.0050 (7)	−0.0003 (7)
C52	0.0222 (10)	0.0206 (10)	0.0197 (10)	0.0000 (8)	0.0051 (8)	−0.0015 (8)
C53	0.0266 (10)	0.0266 (11)	0.0215 (10)	0.0019 (9)	−0.0025 (8)	−0.0014 (9)
C54	0.0420 (12)	0.0200 (10)	0.0118 (9)	−0.0052 (9)	0.0030 (8)	−0.0007 (8)
C55	0.0344 (11)	0.0216 (10)	0.0184 (10)	−0.0075 (9)	0.0125 (8)	−0.0036 (9)
C56	0.0227 (9)	0.0169 (9)	0.0177 (9)	−0.0036 (8)	0.0075 (7)	−0.0013 (8)
C341	0.0241 (10)	0.0173 (10)	0.0423 (13)	0.0024 (8)	0.0124 (9)	−0.0073 (9)

### Geometric parameters (Å, °)

**Table:**

C1—O1	1.2210 (19)	C31—C32	1.399 (2)
C1—C11	1.494 (2)	C32—C33	1.384 (2)
C1—C2	1.516 (2)	C32—H32A	0.9500
C2—C3	1.540 (2)	C33—C34	1.387 (2)
C2—H2A	0.9900	C33—Br33	1.8975 (15)
C2—H2B	0.9900	O34—C34	1.3639 (19)
C3—C31	1.519 (2)	O34—C341	1.427 (2)
C3—C4	1.533 (2)	C34—C35	1.390 (2)
C3—H3A	1.0000	C35—C36	1.384 (2)
C4—C5	1.518 (2)	C35—H35A	0.9500
C4—H4A	0.9900	C36—H36A	0.9500
C4—H4B	0.9900	C51—C52	1.386 (2)
O5—C5	1.2214 (19)	C51—C56	1.398 (2)
C5—C51	1.494 (2)	C52—C53	1.386 (3)
C11—C16	1.394 (2)	C52—H52A	0.9500
C11—C12	1.398 (2)	C53—C54	1.386 (3)
C12—C13	1.382 (2)	C53—H53A	0.9500
C12—H12A	0.9500	C54—C55	1.381 (3)
C13—C14	1.384 (3)	C54—H54A	0.9500
C13—H13A	0.9500	C55—C56	1.386 (2)
C14—C15	1.392 (3)	C55—H55A	0.9500
C14—H14A	0.9500	C56—H56A	0.9500
C15—C16	1.376 (2)	C341—H34D	0.9800
C15—H15A	0.9500	C341—H34A	0.9800
C16—H16A	0.9500	C341—H34B	0.9800
C31—C36	1.382 (2)		
			
O1—C1—C11	120.32 (16)	C36—C31—C3	121.46 (14)
O1—C1—C2	120.49 (15)	C32—C31—C3	120.72 (15)
C11—C1—C2	119.20 (14)	C33—C32—C31	119.98 (15)
C1—C2—C3	114.82 (13)	C33—C32—H32A	120.0
C1—C2—H2A	108.6	C31—C32—H32A	120.0
C3—C2—H2A	108.6	C32—C33—C34	122.15 (15)
C1—C2—H2B	108.6	C32—C33—Br33	118.64 (12)
C3—C2—H2B	108.6	C34—C33—Br33	119.20 (12)
H2A—C2—H2B	107.5	C34—O34—C341	117.10 (13)
C31—C3—C4	109.78 (13)	O34—C34—C33	117.34 (14)
C31—C3—C2	113.04 (13)	O34—C34—C35	125.00 (15)
C4—C3—C2	110.01 (14)	C33—C34—C35	117.66 (15)
C31—C3—H3A	107.9	C36—C35—C34	120.34 (16)
C4—C3—H3A	107.9	C36—C35—H35A	119.8
C2—C3—H3A	107.9	C34—C35—H35A	119.8
C5—C4—C3	114.18 (13)	C31—C36—C35	122.09 (15)
C5—C4—H4A	108.7	C31—C36—H36A	119.0
C3—C4—H4A	108.7	C35—C36—H36A	119.0
C5—C4—H4B	108.7	C52—C51—C56	119.20 (16)
C3—C4—H4B	108.7	C52—C51—C5	122.53 (15)
H4A—C4—H4B	107.6	C56—C51—C5	118.26 (15)
O5—C5—C51	121.14 (15)	C51—C52—C53	120.60 (17)
O5—C5—C4	121.06 (15)	C51—C52—H52A	119.7
C51—C5—C4	117.76 (14)	C53—C52—H52A	119.7
C16—C11—C12	119.09 (16)	C54—C53—C52	119.80 (18)
C16—C11—C1	119.11 (16)	C54—C53—H53A	120.1
C12—C11—C1	121.77 (16)	C52—C53—H53A	120.1
C13—C12—C11	119.63 (18)	C55—C54—C53	120.14 (17)
C13—C12—H12A	120.2	C55—C54—H54A	119.9
C11—C12—H12A	120.2	C53—C54—H54A	119.9
C12—C13—C14	120.94 (18)	C54—C55—C56	120.20 (17)
C12—C13—H13A	119.5	C54—C55—H55A	119.9
C14—C13—H13A	119.5	C56—C55—H55A	119.9
C13—C14—C15	119.63 (17)	C55—C56—C51	120.03 (17)
C13—C14—H14A	120.2	C55—C56—H56A	120.0
C15—C14—H14A	120.2	C51—C56—H56A	120.0
C16—C15—C14	119.69 (19)	O34—C341—H34D	109.5
C16—C15—H15A	120.2	O34—C341—H34A	109.5
C14—C15—H15A	120.2	H34D—C341—H34A	109.5
C15—C16—C11	121.02 (18)	O34—C341—H34B	109.5
C15—C16—H16A	119.5	H34D—C341—H34B	109.5
C11—C16—H16A	119.5	H34A—C341—H34B	109.5
C36—C31—C32	117.75 (15)		
			
O1—C1—C2—C3	109.67 (17)	C31—C32—C33—C34	0.3 (2)
C11—C1—C2—C3	−70.76 (18)	C31—C32—C33—Br33	−179.41 (12)
C1—C2—C3—C31	−62.13 (18)	C341—O34—C34—C33	−170.80 (15)
C1—C2—C3—C4	174.75 (13)	C341—O34—C34—C35	8.7 (2)
C31—C3—C4—C5	160.56 (14)	C32—C33—C34—O34	177.99 (15)
C2—C3—C4—C5	−74.43 (17)	Br33—C33—C34—O34	−2.3 (2)
C3—C4—C5—O5	−2.1 (2)	C32—C33—C34—C35	−1.5 (2)
C3—C4—C5—C51	−179.88 (14)	Br33—C33—C34—C35	178.17 (12)
O1—C1—C11—C16	−3.8 (2)	O34—C34—C35—C36	−177.85 (15)
C2—C1—C11—C16	176.60 (15)	C33—C34—C35—C36	1.6 (2)
O1—C1—C11—C12	174.16 (15)	C32—C31—C36—C35	−0.7 (2)
C2—C1—C11—C12	−5.4 (2)	C3—C31—C36—C35	176.29 (15)
C16—C11—C12—C13	0.1 (2)	C34—C35—C36—C31	−0.5 (3)
C1—C11—C12—C13	−177.90 (16)	O5—C5—C51—C52	179.37 (16)
C11—C12—C13—C14	−0.2 (3)	C4—C5—C51—C52	−2.9 (2)
C12—C13—C14—C15	0.0 (3)	O5—C5—C51—C56	−1.6 (2)
C13—C14—C15—C16	0.2 (3)	C4—C5—C51—C56	176.14 (15)
C14—C15—C16—C11	−0.2 (3)	C56—C51—C52—C53	−1.1 (3)
C12—C11—C16—C15	0.1 (3)	C5—C51—C52—C53	177.88 (16)
C1—C11—C16—C15	178.16 (16)	C51—C52—C53—C54	0.2 (3)
C4—C3—C31—C36	−115.94 (17)	C52—C53—C54—C55	1.2 (3)
C2—C3—C31—C36	120.81 (17)	C53—C54—C55—C56	−1.7 (3)
C4—C3—C31—C32	61.0 (2)	C54—C55—C56—C51	0.7 (3)
C2—C3—C31—C32	−62.3 (2)	C52—C51—C56—C55	0.7 (3)
C36—C31—C32—C33	0.8 (2)	C5—C51—C56—C55	−178.35 (15)
C3—C31—C32—C33	−176.20 (15)		

### Hydrogen-bond geometry (Å, °)

**Table:**

*Cg*1 is the centroid of the C31–C36 ring.

**Table:**

*D*—H···*A*	*D*—H	H···*A*	*D*···*A*	*D*—H···*A*
C13—H13A···Br33^i^	0.95	2.76	3.613 (2)	149
C35—H35A···O1^ii^	0.95	2.37	3.245 (2)	153
C36—H36A···O5^iii^	0.95	2.56	3.493 (2)	168
C54—H54A···Cg1^iv^	0.95	2.60	3.489 (3)	155

Symmetry codes: (i) *x*+1, *y*, *z*; (ii) *x*, *y*+1, *z*; (iii) −*x*+2, *y*+1/2, −*z*+1/2; (iv) *x*, −*y*+1/2, *z*−1/2.
